# Own-Group Face Recognition Bias: The Effects of Location and Reputation

**DOI:** 10.3389/fpsyg.2017.01734

**Published:** 2017-10-10

**Authors:** Linlin Yan, Zhe Wang, Jianling Huang, Yu-Hao P. Sun, Rebecca A. Judges, Naiqi G. Xiao, Kang Lee

**Affiliations:** ^1^Department of Psychology, Zhejiang Sci-Tech University, Hangzhou, China; ^2^Department of Applied Psychology and Human Development, University of Toronto, Toronto, ON, Canada; ^3^Department of Psychology, Princeton University, Princeton, NJ, United States

**Keywords:** own-group face recognition bias, in-group members, out-group members, location, reputation

## Abstract

In the present study, we examined whether social categorization based on university affiliation can induce an advantage in recognizing faces. Moreover, we investigated how the reputation or location of the university affected face recognition performance using an old/new paradigm. We assigned five different university labels to the faces: participants’ own university and four other universities. Among the four other university labels, we manipulated the academic reputation and geographical location of these universities relative to the participants’ own university. The results showed that an own-group face recognition bias emerged for faces with own-university labels comparing to those with other-university labels. Furthermore, we found a robust own-group face recognition bias only when the other university was located in a different city far away from participants’ own university. Interestingly, we failed to find the influence of university reputation on own-group face recognition bias. These results suggest that categorizing a face as a member of one’s own university is sufficient to enhance recognition accuracy and the location will play a more important role in the effect of social categorization on face recognition than reputation. The results provide insight into the role of motivational factors underlying the university membership in face perception.

## Introduction

People treat in-group and out-group members differently. One example of this robust phenomenon is own-race face recognition bias (see Meissner and Brigham, 2001 for review and meta-analysis), that is, own-race faces are better recognized than other-race faces. Given the salience of the racial features contained in the face, it is no surprise that preferred attention and cognitive resources are allocated toward the individual’s category membership and inter-group bias exists. However, superior recognition for one’s in-group as compared to an out-group might occur even without salient physiognomic facial features, such as group based on social economic status (Shriver et al., 2008), religious belief (Rule et al., 2010), political orientation (Ray et al., 2010), and university affiliation (Bernstein et al., 2007).

Recently, a series of own group face recognition bias have been consistently demonstrated for targets with own/other university labels (Shriver et al., 2008; Hehman et al., 2010; Van Bavel et al., 2012; Ng et al., 2016). Compared to faces belonging to the other university, people more efficiently encode (Cassidy et al., 2011) and better recognize faces that are labeled with the same university. One influential hypothesis about own-group face recognition bias is the social cognitive model, which proposes that people are motivated to individuate in-group members but categorize out-group members (Sporer, 2001; Hugenberg et al., 2010, 2013). And as such, the motivational nature of the social cognitive model predicts that virtually any contextually meaningful shared in-group membership may signal the need to individuate. Thus, it is conceivable that undergraduates are treating the faces with their own university label as in-group members and better recognizing them than the faces with other university label. However, little is known about the social motivational underpinnings of the own-group bias manipulated by the university membership.

Note that social reputation of the university may be one of the most important motivational factors that might affect the undergraduates’ evaluation of the universities and enhance the memory of targets from prestigious universities. Snibbe et al. (2003) found that both Kyoto and Ritsumeikan students in Japan favored Kyoto University (better reputation) over Ritsumeikan University, reflecting the undergraduates’ focus on the reputation of the universities. Targets from top-ranked universities might signal diligence and professionalism and the perceivers might favor targets from prestigious universities over universities with poor reputation. The reputation difference between own-university and other-university did exist in most studies mentioned above (Miami and Marshall University in the United States, Bernstein et al., 2007; Shriver et al., 2008; University of Delaware and James Madison University in United States, Hehman et al., 2010) and mostly better reputation of own-university than other-university. For example, Miami university (own university, 656th) is ranked significantly higher than Marshall university (other university, 1287th) according to Ranking Web of Universities in the world ^1^. Furthermore, Van Bavel et al. (2012) found no own-group face recognition bias between Ohio State University and University of Toronto amongst Ohio State students. This might be due to the fact that University of Toronto (other university, 18th) is consistently ranked higher than Ohio State University (own university, 33rd) on a number of international reputation rankings, and thus participants may have focused more on the relatively higher status out-group. Therefore, higher reputation of the own university might be the primary cause that enhance the memory of in-group targets and elicit the own-group bias.

However, higher reputation of the own university might not be the only cause that elicits the own-group recognition bias manipulated by university labels. For example, Simon Fraser University (other university, 119th) is ranked higher than York university (own university, 221st) according to Ranking Web of Universities in the world. However, Ng et al. found that Canadian participants showed enhanced memory of targets from their own university with a poorer reputation than another university. It is worth noting that the two universities are located separately in the east and west of Canada with a spatial distance of 4227 kilometers. Abbott and Schmid (1975) demonstrated that undergraduates favored prestigious university in the east of the United States, but not favored prestigious university in the west. And Snibbe et al. (2003) found significantly less in-group favoritism among Japanese undergraduates compared with their American counterparts. This may be because the own and the other universities in Japan were located at the same city of Kyoto but both American universities were separately located at the west coast and the north central of the United States. Therefore, geographical location might be another important motivational factor that might affect the undergraduates’ evaluation of the universities and elicit the own-group bias. Spatial distance from a target determines the amount and kind of information that is available about the target (Liberman et al., 2007). As one may be closer to the targets from the same-location university than the different-location university, they might be motivated to process the information of university members from the same location with more accuracy and detail. This could be why targets from the own university are better recognized than other university. To date, no studies have directly explored the contribution made by the motivational factors (reputation and location) underlying the social group membership (university affiliation).

Given that China has a number of universities differing significantly in reputation and geographical location, and that Chinese undergraduates shared identities based on university affiliation, we speculated social categorization manipulated with the university labels might elicit an inter-group face memory bias in China. We aimed to explore whether reputation or location affect the own-group face recognition bias in Chinese undergraduates. Specifically, we selected undergraduates from Zhejiang Sci-Tech University (ZSTU) as participants and four other university affiliations with different reputations and locations in China as out-group membership labels. According to the map of China, Hangzhou Dianzi University (HZDU) and Zhejiang University (ZJU) are located in the same city of Hangzhou as the participants’ own university, but Taiyuan University of Technology (TYUT) and Tsinghua University (TSU) are located in the north of China and far away from participants’ own university. Meanwhile according to the Ranking Web of Universities in China^2^, ZJU (3rd) and TSU (1st) are obviously ranked higher than the participants’ own university (ZSTU, 111th), but both HZDU (124th) and TYUT (104th) are ranked similarly as participants’ own university. Therefore, when comparing university affiliations (same location/same reputation, same location/different reputation, different location/same reputation and different location/different reputation), we examined the effects of reputation and location of the university on the own-group face recognition bias.

If the location plays an important role in the own-group bias, then one would expect poorer recognition performance for the faces labeled with other university from a different location as one’s own university (indicated as own-group face recognition bias), and no or very little difference in recognition performance between the faces labeled with the own university and other university from the same location (indicated as no own-group face recognition bias). If the reputation plays an important role in the social categorization, then one would expect better recognition performance of the faces labeled with the other university ranked higher than one’s own university (indicated as other-group face recognition bias), and no or very little difference in recognition performance between the faces labeled with the own university and other university with the same ranking.

## Materials and Methods

### Subjects and Design

One hundred and fifty Chinese undergraduates (59 males, mean age = 20.3 years, *SD* = 1.4) from ZSTU took part in the study, separated randomly into no label group and the four different own-other university labeled groups [ZSTU-HZDU (Hangzhou Dianzi University): same location and same reputation; ZSTU-ZJU (Zhejiang University): same location and different reputation; ZSTU-TYUT (Taiyuan University of Technology): different location and same reputation; ZSTU-TSU (Tsinghua University): different location and different reputation] groups. The no label group was included as a control group for two reasons: (1) to exclude the possible effect of the color background on the face recognition performance; and (2) to confirm how including a university label would affect face recognition performance. All participants had normal or corrected-to-normal vision, were right-handed based on self-report and were paid for participation. This study was carried out in accordance with the recommendations of the Human Research Ethics Committee of Zhejiang Sci-Tech University with written informed consent from all subjects. Participants gave written informed consent according to Declaration of Helsinki prior to their participation, and the protocol was approved by the Human Research Ethics Committee of ZSTU. A 2 (location: universities with same location vs. universities with different locations) × 2 (reputation: universities with same reputation vs. universities with different reputations) between-subject experimental design was used for the university labeled condition.

### Stimuli

**FIGURE 1:**
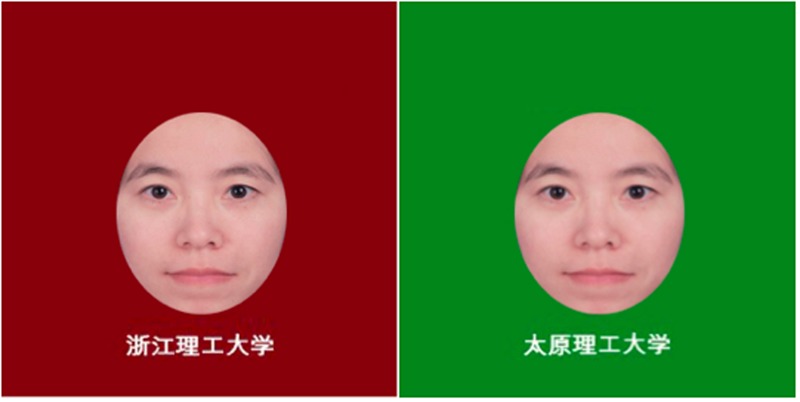
Examples of learning phase stimuli (left: ZSTU; right: TYUT). The authors received signed consent from individuals to have their photos taken and reproduced for the research.

Sixty Chinese hairless full-front faces in color were used as the stimuli (30 female, no stimuli were ZSTU, HZDU, ZJU, TYUT or TSU students, from the face pool of Kang Lee’s lab), unfamiliar to the participants, posing with a neutral expression. Adobe Photoshop was used to edit the images to get rid of specific features (i.e., mole) and resize them to approximately 4.5 cm × 5.5 cm. The sixty faces were assigned into two lists (15 female in each list). One list is used for red background and the other is used for green background. The two lists of the faces were counterbalanced between participants. For no label group, there was no extra information about the faces on the red/green background. For university label group (**Figure 1**), the university name (ZSTU for red background; HZDU, ZJU, TYUT, and TSU for green background) was inscribed in white Chinese characters (i.e., 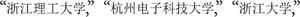 “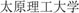,” and “
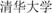
”) at the bottom of the background. For the participants in the label group, the faces presented against red background were indicated as in-group and those faces presented against green background were indicated as out-group.

### Procedure

After providing informed consent, participants were instructed to complete an old/new face recognition task consisting of a learning phase and a recognition phase. All instructions and stimuli were presented with E-Prime 2.0 (Psychology Software Testing, Pittsburgh, PA, United States) via a computer. Before the learning phase, participants were instructed that they would see 30 faces on the computer screen and should attend closely to these faces in order to recognize them later. Participants were instructed that they would see the university labels below the faces against red/green background (the faces on red background were fellow ZSTU students, whereas the faces on green background were other university students, i.e., HZDU, ZJU, TYUT, or TSU separately). However, participants in the no label group received no specific instructions regarding university categorization but the background colors categorization. During the learning phase, each trial started with a fixation cross (500 ms), followed by a target face with a red or green background appearing for 2000 ms, and the interstimulus interval for 500 ms. After 30 faces (half on the red background, the other half on the green background) are randomly presented on the computer screen, the participants were instructed to rest for 3–5 min. Then during the recognition phase, the participants were instructed that they would see a series of faces, some of which they had seen (i.e., old faces) during the learning phase and some of which they had not seen before (i.e., new faces). Participants were instructed to decide as accurately as possible whether the target face was seen or not (left or right keys, counterbalanced across participants) with a maximum display time of 2000 ms. If participants did not respond within 2000 ms after the onset of the face, the face will disappear and the trial will not be brought into data analysis. All the 60 faces presented against corresponding red/green background randomly during the recognition phase included the 30 faces previously seen during learning phase and 30 new faces without university labels. Before the formal experimental trials, participants were asked to be familiar with the procedure through simple old/new object recognition. After completing all the tasks, participants in the label groups were asked to sort the five universities in a descending order according to their reputations in China (i.e., 5 indicated ranking first). In addition, participants were also asked to report the cities where the other four universities located, and sort them in a descending order according to the distance between them and ZSTU (i.e., 5 indicated longest distance). After completing all tasks, participants were probed for suspicion, thanked, and debriefed.

## Results

From the self-report of the participants in the label groups, we’ve found that they reported the accurate cities the five universities located and sorted them accurately according to the distance between other universities and their own university. Furthermore, we have found that the participants sorted the universities according to their reputations in China. The higher score a university receives, the higher rank it is among the five universities. According to the participants’ reputation ratings, we found that almost all the participants ranked TSU (*M* = 4.9, *SE* = 0.028) as the most prestigious university, ZJU (*M* = 4.1, *SE* = 0.028) as the second prestigious university and TYUT (*M* = 1.62, *SE* = 0.074) as the last place, all *ps* < 0.001. And ZSTU (*M* = 2.14, *SE* = 0.077) and HZDU (*M* = 2.25, *SE* = 0.059) were similar in ranking, *p* = 0.35. As we expected, the rating scores suggest that all the participants knew the different reputation associations with the respective universities.

Of interest was the extent to which the presence of the university labels influenced face recognition. Thus, measures of sensitivity (*d′ = z* [hits] *– z* [FA]) and response bias (*C* = -0.5 [*z* (hits) + *z* (FA)]) were computed according to signal detection theory (Green and Swets, 1966), which can be created from hit rates (the correct identification of an old face) and false alarm rates (the misidentification of a new face as an old face) separately for targets with own-university and other-university labels (indicated in **Table 1**). For the no label group, *d′* and *C* were calculated separately for targets on red and green backgrounds. If there were any hit and false-alarm rates of 100 or 0%, we did a standard correction replacing 100 and 0% with ‘1-1/2N’ and ‘1/2N’ separately, where N is the maximum number of targets (Macmillan and Kaplan, 1985).

**Table 1:** *d′* and *C* (standard deviation for faces with own and other universities as function of category label.

Group	Labels	*d′* (SE)	*C* (SE)
No label	Red	0.28 (0.09)	-0.001 (0.06)
	Green	0.43 (0.11)	-0.028 (0.06)
Same location/same reputation	ZSTU	0.71 (0.11)	0.007 (0.07)
	HZDU	0.68 (0.12)	0.036 (0.06)
Same location/different reputation	ZSTU	0.63 (0.11)	-0.019 (0.06)
	ZJU	0.72 (0.13)	-0.046 (0.06)
Different location/same reputation	ZSTU	0.83 (0.09)	-0.159 (0.12)
	TYUT	0.34 (0.12)	-0.151 (0.07)
Different location/different reputation	ZSTU	0.70 (0.12)	0.065 (0.08)
	TSU	0.47 (0.10)	0.004 (0.05)

*Values are represented as means (standard error).*

All trials lasting 2000 ms or less were included (no label group: 60 ± 0.00; TYUT-ZSTU: 59 ± 0.08; TSU-ZSTU: 59 ± 0.14; HZDU-ZSTU: 59 ± 0.08; ZJU-ZSTU: 59 ± 0.09) in the analyses. The preliminary analyses have confirmed the face assignment (*p* = 0.48) and face gender (*p* = 0.38) did not influence face recognition performance. As expected, there was a main effect of the label [*F*(4,140) = 8.27, *p* < 0.01, $\eta_{p}^{2}$ = 0.19]. The *post hoc* analysis demonstrated that the no label group was worse at recognizing faces than another four labeled groups, *ps* < 0.01, and there were no difference between labeled groups, all the other *ps* > 0.65. In addition, a significant interaction emerged between participant gender and target face gender, *F*(1,148) = 4.72, *p* = 0.031, $\eta_{p}^{2}$ = 0.031, i.e., better memory for faces of one’s own gender (own-gender bias, Herlitz and Lovén, 2013). Except that, there were no other significant interactions, all the other *ps* > 0.24. Therefore, we decided not to include these factors in the following data analyses.

**FIGURE 2:**
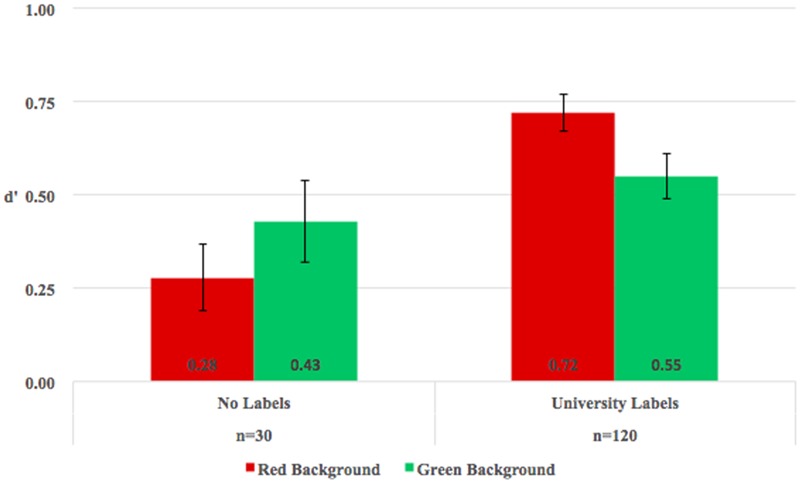
Recognition accuracy (*d′*) for faces with university labels and without labels. Under the condition of University labels, red bars refer to own university (ZSTU); green bars refer to other university. Error bars represent standard error.

To test whether the presence of university labels influenced face recognition, we conducted a 2 (background color: red vs. green) × 2 (university label: no label vs. label) mixed-model analysis of variance (ANOVA), with repeated measures on the first factor. The ANOVA results (see **Figure 2**) revealed a main effect of university label [*F*(1,148) = 7.70, *p* = 0.006, $\eta_{p}^{2}$ = 0.05] and no main effect of background color [*F*(1,148) = 0.008, *p* = 0.93, $\eta_{p}^{2}$ < 0.001]. Faces labeled with university affiliations (*M* = 0.63, *SE* = 0.04) were better recognized than faces without labels (*M* = 0.35, *SE* = 0.09). And the interaction between background color and university label was significant, *F*(1,148) = 4.98, *p* = 0.03, $\eta_{p}^{2}$ = 0.03. When no university labels were present, face recognition performance was equivalent for the red (*M* = 0.28, *SE* = 0.09) and green (*M* = 0.43, *SE* = 0.11) backgrounds, *t*(29) = -1.21, *Cohen’s d* = 0.22, *p* = 0.24. However, when university labels were present, faces on the red backgrounds (i.e., in-group members; *M* = 0.72, *SE* = 0.05) were better recognized than were faces on the green background (i.e., out-group members; *M* = 0.55, *SE* = 0.06), *t*(119) = 2.59, *Cohen’s d* = 0.24, *p* = 0.01. When participants did not believe the background color was signaling group membership, no recognition bias emerged. When background was indicative of university membership, in-group faces were better recognized than out-group faces. Furthermore, when the university label was other university (i.e., green background), face recognition performance was equivalent with and without label, *F*(1,148) = 0.88, *p* = 0.35, $\eta_{p}^{2}$ = 0.01. When the university label was own university (i.e., red background), faces were better recognized with label than without label, *F*(1,148) = 14.26, *p* < 0.001, $\eta_{p}^{2}$ = 0.09. Therefore, the presence of university labels influenced the face recognition and the enhanced recognition of faces labeled with own-university elicited own-group bias.

**FIGURE 3:**
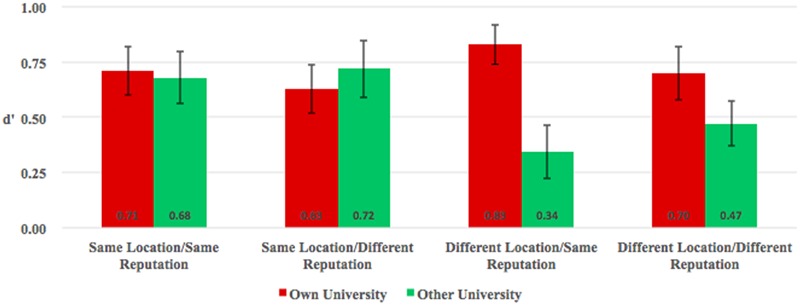
Recognition accuracy (*d′*) for faces with own and other universities as function of category label. Red bars refer to own university (ZSTU); green bars refer to other university (HZDU, ZJU, TYUT, TSU, separately). Error bars represent standard error.

To explore whether the group membership and motivational factor (reputation or location) would affect the own-group face recognition bias in Chinese undergraduates, we conducted a 2 (group: in-group vs. out-group) × 2 (reputation: same vs. different) × 2 (location: same vs. different) mixed-model analysis of variance (ANOVA) on *d′* measure, with repeated measures on the first factor. Results (**Figure 3**) showed no main effect of location [*F*(1,116) = 1.09, *p* = 0.3, $\eta_{p}^{2}$ = 0.009] and reputation [*F*(1,116) = 0.005, *p* = 0.94, $\eta_{p}^{2}$ < 0.001], but a main effect of group [*F*(1,116) = 7.28, *p* = 0.008, $\eta_{p}^{2}$ = 0.059]. The results demonstrated that recognition performance was better for in-group (*M* = 0.72, *SE* = 0.05) than out-group (*M* = 0.55, *SE* = 0.06). And the interaction between group and location was significant, *F*(1,116) = 10.49, *p* = 0.002, $\eta_{p}^{2}$ = 0.083. The follow-up analysis (**Figure 3**) showed that, when the labeled universities are from the same location, recognition performance was equivalent for in-group university and out-group university, no matter the reputation of other university is higher [ZSTU: *M*_own_ = 0.63, *SE*_own_ = 0.11; ZJU: *M*_other_ = 0.72, *SE*_other_ = 0.13; *t*(29) = -0.79, *Cohen’s d* = 0.15, *p* = 0.43] or equal [ZSTU: *M*_own_ = 0.71, *SE*_own_ = 0.11; HZDU: *M*_other_ = 0.68, *SE*_other_ = 0.12; *Cohen’s d* = 0.04, *t*(29) = 0.20, *p* = 0.84] to own university. However, when the labeled universities are from different locations, recognition performance was better for in-group university than out-group university, no matter the reputation of the other university is higher [ZSTU: *M*_own_ = 0.70, *SE*_own_ = 0.12; TSU: *M*_other_ = 0.47, *SE*_other_ = 0.10; *t*(29) = 1.85, *Cohen’s d* = 0.34, *p* = 0.07] or equal [ZSTU: *M*_own_ = 0.83, *SE*_own_ = 0.09; TYUT: *M*_other_ = 0.34, *SE*_other_ = 0.12; *t*(29) = 3.87, *Cohen’s d* = 0.71, *p* = 0.001] to own university. However, there were not any other significant interactions, all *ps* > 0.12. Furthermore, we calculated the ranking evaluation difference and the *d′* difference between other university and own university separately: ΔReputation = R_other_ – R_own_ and Δ*d′* = *d′*_other_ – *d′*_own_. The Pearson correlation analysis demonstrated that there was a marginal significant correlation between the ΔReputation and Δ*d′* (*r* = 0.16, *p* = 0.08). The results suggested that other university labels signaling different geographical locations from one’s own university is sufficient to elicit own-group face recognition bias. Whereas other university labels signaling a different reputation from the own university might play a certain role in face recognition performance, but it was not sufficient to exceed the role of the location in own-group recognition bias. Therefore, the location between the own university and other university is not only sufficient but also necessary to elicit the own-group face recognition bias in Chinese undergraduates.

The second parameter C reflects a bias in participants’ response (e.g., a tendency to report “yes”). To explore whether the group membership and motivational factor (reputation and location) affected the response bias, we conducted a 2 (group: in-group vs. out-group) × 2 (reputation: same vs. different) × 2 (location: same vs. different) mixed ANOVA on participants’ the response bias: C. The results showed no significant main effects or interactions, *ps* > 0.27, except a marginal interaction between location and reputation, *F*(1,116) = 3.95, *p* = 0.05, $\eta_{p}^{2}$ = 0.03. The follow-up analysis demonstrated that when the own and other university were similar in reputation, participants tended to respond marginally more liberally for the faces from the university of different locations (*M* = -0.16, *SE* = 0.45) compared with the university of same location (*M* = 0.02, *SE* = 0.29), *p* = 0.08; when the own and other university were different in location, participants tended to respond marginally more liberally for the faces from the university of same reputation (*M* = -0.16, *SE* = 0.45) compared with the university of different reputation (*M* = 0.03, *SE* = 0.33), *p* = 0.08. Other than these marginal patterns, there were no other significant effects, all the other *ps* > 0.27. The results suggested that almost all the participants showed no specific strategies, except for a marginal tendency to report remembering faces from the university of different locations/same reputation.

## Discussion

In the current study, we found that social categorization manipulated with university labels elicits an own-group face recognition bias. More than that, by manipulating the locations and reputations of the other university labels we found that the geographic location of the other university plays a more important role in own-group bias than reputation. That is, when the other university labels signaled a different geographical location from one’s own university, it is sufficient to elicit own-group face recognition bias; but if other university labels signaled a different reputation from the own university is not sufficient, even when the other university is ranked higher than one’s own-university. Therefore, it appears that categorizing a face as a member of one’s own university is sufficient to enhance recognition accuracy and the geographic location will take priority as a social membership over reputation.

Consistent with previous evidence of the own-university face recognition bias (Bernstein et al., 2007; Hehman et al., 2010; Van Bavel et al., 2012), merely categorizing faces as belonging to one’s own university facilitated their recognition, relative to faces believed to belong to another university. Specifically, this phenomenon occurred even though group distinction might be ambiguous from the perceptual markers of faces. Why did the own-group recognition bias exist even when the visual features distinguishing the groups were unclear? According to the social cognitive model, own-group bias can emerge because perceivers categorize out-group members but are motivated to individuate in-group members (Hugenberg et al., 2010). The shared university membership might be an external motivating factor to individuate targets and better recognize them later. As our results indicated, faces with labels signaling one’s own-university members could be better recognized compared to faces without the label; in contrast, faces with the label signaling other university members were not better recognized compared to faces without the label. However, in other studies no such bias emerged in situations where participants or targets held multiple identities and category memberships (Shriver et al., 2008; Ng et al., 2016), such as racial membership and university membership. Given that individuals hold multiple identities and category memberships, group membership was subject to the whims of the situation (Hogg and Turner, 1987). As a perceiver’s salient identity shifts, an out-group member in one situation may be perceived as in-group in another (Hugenberg and Bodenhausen, 2004). The participants and targets in our study were of same race and the stimuli were counterbalanced across conditions. Therefore, the university membership was the only salient group distinction and elicited the own-group face recognition bias in Chinese undergraduates. In short, own-group face recognition bias emerged in situations where the group distinction was obvious.

Furthermore, it is important to note that the own-university face recognition bias may appear quite fickle across situations, depending on the motivational factors (location and reputation) underlying the university membership. Our results indicated that the poorer recognition performance for the faces labeled with another university from a different location (i.e., TYUT and TSU located in the North far away from ZSTU) as one’s own university, and no difference in recognition performance between the faces labeled with the own university and an other university (i.e., HZDU and ZJU located in the same city as ZSTU) from the same location, no matter of the reputation difference between own university and an other university. People vary in their valuations of different groups, and the psychological significance of group membership is a powerful moderator of their behaviors toward in-group and out-group members (Van Bavel et al., 2012). The spatial distance between the own and other university might play a more important role in social categorization than the reputation in Chinese undergraduates. It is possible that the undergraduates are likely to be more connected with students at the nearby university than another university far away. For example, undergraduates who are geographically closer together often have a greater chance of being one’s classmate or work colleague than those who attend universities far away. Surprisingly, we did not find significant evidence that the higher reputation of the other university than one’s own university would facilitate the memory of targets. One possibility is that participants often have limited chance to interact with the targets from universities of higher reputation and might not be motivated to individuate them. Another possibility is that participants may have focused more attention on the location distinction than reputation distinction. The geographical location information may be more perceptually obvious from the university labels than reputation information. Although more studies are needed to investigate these possibilities, the current research provides novel evidence that geographical location distinctions underlying university membership are sufficient to elicit differences in face recognition and take priority to be processed than reputation distinction.

The group-based bias in face recognition reflects specific social categorization, which is largely shaped by environmental factors, such as culture (Ng et al., 2016). Different cultural context may emphasize different dimensions when it comes to social categorization (Yuki and Brewer, 2014). The current finding that geographic location triumphs over reputation in affecting the own-group face recognition bias of Chinese undergraduates may be a cultural specific phenomenon. In East Asian cultures, social groups are more likely to be conceived of as networks of interpersonal relationships (Brewer and Yuki, 2007), which are largely represented by geographic distributions. When the geographic distance between the two universities was very close (e.g., in the same city), the tight-knit social relations made the boundaries between in-group and out-group difficult to distinguish. Therefore, we’ve found that a similar enhanced face recognition performance emerged when the other university was located in the same city of participants’ own university. In contrast, in Western cultures, the inclination of being stationary in a place for generations is much weaker compared to that in Eastern Asian cultures. Thereby, people in Western countries might be less likely to use geographic distance to represent the strength of social connections, and geographic location may be less likely to affect own-group face recognition performance. In line with this cultures specificity hypothesis, Kealy and Rockel (1987) studied American students’ perceptions of the university’s quality on four dimensions (academic reputation, social atmosphere, location of campus and athletic quality) and found that the students were predisposed toward athletic quality and academic reputation. Yang et al. (2008) also found that the American students focused on the academic reputation and the sports program. Therefore, the reputation might play a more important role than location in Western cultures. To confirm the roles of culture on the own-group face recognition bias based on the university membership, a cross-culture design is necessary for future studies.

In summary, the present study contributes to research examining face perception by demonstrating the effects of location and reputation underlying university membership on the face recognition bias. Person memory is modulated not only by social categorization but also by the psychological significance of the social category. Overall, our results provide additional evidence that social categorization and motivational processes can affect face recognition bias; social distance constitutes more meaningful in-groups than university reputation in China.

## Author Contributions

LY: designing study, recruitment, data collection, analyzing results, writing final manuscript. ZW: designing study, analyzing results, writing final manuscript. JH: recruitment, data collection. Y-HS: analysis support, writing final manuscript. RJ: writing final manuscript. NX: writing final manuscript. KL: designing study, analyzing results, writing final manuscript.

## Conflict of Interest Statement

The authors declare that the research was conducted in the absence of any commercial or financial relationships that could be construed as a potential conflict of interest.

## Footnotes

1 http://www.webometrics.info/en/world (July 2017 Edition)

2 http://www.webometrics.info/en/Asia/China%20, July 2017 Edition
